# Current status and influencing factors of prophylactic use of proton pump inhibitors in internal medicine inpatients receiving glucocorticoid therapy

**DOI:** 10.3389/fphar.2024.1418086

**Published:** 2024-09-04

**Authors:** Pengpeng Liu, Guangyao Li, Qian Wu, Mei Han

**Affiliations:** ^1^ Department of Pharmacy, Beijing Tongren Hospital, Capital Medical University, Beijing, China; ^2^ Department of Neurology, Beijing Tongren Hospital, Capital Medical University, Beijing, China; ^3^ Evidence-Based Medicine Center, Beijing University of Chinese Medicine, Beijing, China

**Keywords:** internal medicine inpatients, glucocorticoids, proton pump inhibitors, prophylaxis, current status, influencing factors

## Abstract

**Background:**

The actual situation and influencing factors of prophylactic use of proton pump inhibitors (PPIs) in internal medicine inpatients receiving glucocorticoid therapy are rarely reported. This study aimed to investigate the current status and influencing factors of prophylactic use of PPIs in internal medicine inpatients receiving glucocorticoid therapy to provide a basis for rational prophylactic use of PPIs.

**Methods:**

Internal medicine inpatients receiving glucocorticoid therapy from February 2023 to September 2023 were included. Information on the prophylactic use of PPIs was collected and analyzed by clinical pharmacists. Associated factors with prophylactic use of PPIs were analyzed by univariable and multivariable logistic regression.

**Results:**

980 inpatients were finally included in our study, of which 271 (27.7%) inpatients received prophylactic use of PPIs. Among the inpatients prescribed PPIs, 90 inpatients received a standard dose of PPIs twice a day. Multiple logistic regression analysis showed that age ≥80 years [OR = 7.009, 95% CI (1.424, 34.495), *p* = 0.017], history of gastroesophageal reflux disease (GERD) [OR = 2.047, 95% CI (1.338, 3.133), *p* = 0.001], low platelet count [OR = 0.997, 95% CI (0.994, 0.999), *p* = 0.004], number of concomitant diseases [OR = 1.104, 95% CI (1.056, 1.153), *p* < 0.001], junior doctors [OR = 1.755, 95% CI (1.248, 2.468), *p* = 0.001], glucocorticoid dose (higher than 50 mg, measured by methylprednisolone) [OR = 2.455, 95% CI (1.371, 4.395), *p* = 0.003], antiplatelet agents [OR = 2.567, 95% CI (1.456, 4.524), *p* = 0.001], immunosuppressants [OR = 1.477, 95% CI (1.014, 2.153), *p* = 0.042], and betahistine [OR = 5.503, 95% CI (1.124, 26.950), *p* = 0.035] were associated with more prophylactic use of PPIs.

**Conclusion:**

The prophylactic use of PPIs in internal medicine inpatients receiving glucocorticoid therapy is common in China. Clinical pharmacists will take targeted measures to promote the rational use of PPIs according to the results of this study.

## 1 Introduction

Glucocorticoids have been widely used in the treatment of various diseases since their clinical application in the 1950s due to their anti-inflammatory, immunosuppressive, anti-allergic, anti-shock, and other pharmacological effects (Vandewalle et al., 2018). Unfortunately, systemic use of glucocorticoids is often associated with multiple adverse reactions, and gastrointestinal complications are one of the adverse reactions (Oray et al., 2016). Therefore, clinicians routinely prescribe acid suppressants or gastric mucosal protectants (GMPs) to prevent glucocorticoid-related gastrointestinal adverse reactions. Proton pump inhibitors (PPIs), acting on H+/K + ATPase (the proton pump), inhibit the last channel of gastric acid secretion and have a good inhibitory effect on basal and food-stimulated acid secretions (Savarino et al., 2017). In recent years, the use of PPIs to prevent glucocorticoid-related gastrointestinal adverse reactions has become increasingly common, and clinical abuse is also common (Savarino et al., 2018). Excessive prophylactic use of PPIs leads to economic and safety issues, so it is necessary to regulate the rational use of PPIs (Savarino et al., 2018).

At present, the relevant guidelines at domestic and foreign suggest that PPIs can be used to prevent stress-related mucosal diseases when the body uses high-dose glucocorticoids (higher than 50 mg, measured by methylprednisolone) while combining with one of the serious risk factors in severe stress conditions such as severe trauma, complex surgery, and critical illness (American Society of Health-System Pharmacists, 1999; National Health Commission of the People’s Republic of China, 2020; Hospital Pharmacy Committee of Chinese Pharmaceutical Association, 2020). Severe stress usually occurs in patients in the emergency department, surgery, and intensive care unit. However, whether PPIs can be used prophylactically in internal medicine inpatients receiving glucocorticoids remains to be considered. By searching the literature, we found that many experts have suggested that whether these glucocorticoid users can use PPIs depends mainly on the risk of gastric mucosal damage, such as those using nonsteroidal anti-inflammatory drugs (NSAIDs), patients with a history of gastrointestinal bleeding, but there is currently a lack of objective evaluation criteria for prophylactic use of PPIs in internal medicine inpatients receiving glucocorticoids (Writing Group of Expert Consensus on the Preventive Application of Proton Pump Inhibitors, 2018; Liu et al., 2013; Caplan et al., 2017). Up to now, few studies have been reported on the actual situation of prophylactic use of PPIs in internal medicine inpatients receiving glucocorticoids in clinical practice.

Therefore, the primary objective of this study was to investigate the current status of prophylactic use of PPIs in internal medicine inpatients receiving glucocorticoids. The second objective was to identify associated factors with the prophylactic use of PPIs. This study will help to raise awareness among internal medicine physicians about the appropriate prophylactic use of PPIs, and also provide supporting information for the development of evaluation criteria for the prophylactic use of PPIs in internal medicine inpatients receiving glucocorticoids in the next step.

## 2 Methods

### 2.1 Setting and study design

This retrospective study was conducted in the Beijing Tongren Hospital affiliated with Capital Medical University, a 1759-bed tertiary care, teaching, and research institution. Patients receiving glucocorticoids during hospitalization in the department of neurology, rheumatology, endocrinology, and nephrology from February 2023 to September 2023 were included. Patients receiving glucocorticoids for infectious diseases, allergic diseases, tumors, asthma, and surgery were excluded. Patients who were prescribed acid suppressants or GMPs for the treatment of gastrointestinal diseases such as gastrointestinal hemorrhage, peptic ulcer (PU), and gastroesophageal reflux disease (GERD) were excluded. Patients with incomplete data were also excluded. The study was approved by the Beijing Tongren Hospital Ethics Committee (NO. TREC2024-KY065). Patients were exempt from informed consent.

### 2.2 Data collection

The following information from the electronic medical records of Beijing Tongren Hospital was collected: demographics (age and gender), smoking and drinking habits, body mass index (BMI), serum creatinine, international normalized ratio (INR), platelet count, types of medical insurance, concomitant diseases, number of concomitant diseases, length of hospital stay, types of medications used, glucocorticoid dose, duration of glucocorticoid therapy, PPIs (drug name, dosage form, usage and dosage, duration, professional titles of doctors).

### 2.3 Statistical analysis

Patients who were prescribed acid suppressants or GMPs were divided into PPIs and non-PPIs groups based on whether or not using PPIs. A descriptive analysis was performed on the patient’s demographics, smoking and drinking habits, BMI, serum creatinine, INR, platelet count, types of medical insurance, comorbidity conditions, number of comorbidities, length of hospital stay, types of medications used, glucocorticoid dose, duration of glucocorticoid therapy, PPIs (drug name, dosage form, usage and dosage, duration, professional titles of doctors).

For continuous variables, the Student’s t-test or the Mann-Whitney *U* test was used to compare the two groups. Categorical variables were described by frequencies and percentages, and between-group differences were analyzed using the Chi-square test and Fisher’s exact test if necessary. Variance inflation factor (VIF) values were calculated to measure the degree of multicollinearity among the variables that were significant in the univariate analysis (*p* < 0.1). A VIF of >10 was considered indicative of multicollinearity and excluded from the logistic regression analysis. Based on the univariate analysis and VIF values, significant variables (*p* < 0.1) were included in the multiple logistic regression analysis to identify influencing factors associated with the prophylactic use of PPIs. All statistical analyses were carried out using SPSS (Version 26.0). P values <0.05 were considered statistically significant.

## 3 Results

### 3.1 Patient characteristics

**FIGURE 1:**
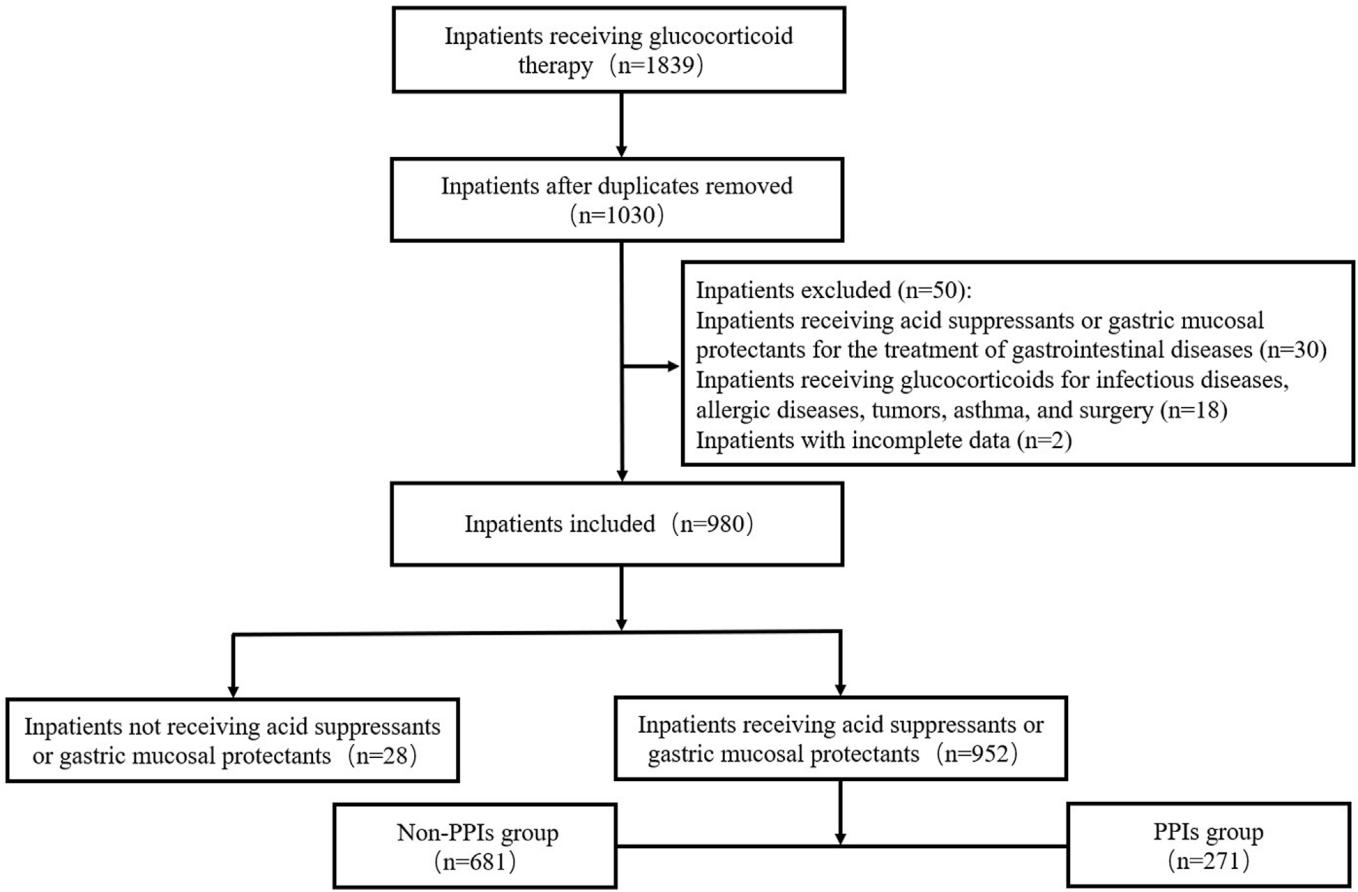
Flowchart of the patient selection process. *PPIs* Proton pump inhibitors.

During the study period, a total of 980 inpatients were finally included in our study. The procedure of patient selection is presented in Figure 1. The median age of the patients was 52.0 (40.0, 60.0) years and the majority (53.8%) were female. The number of these patients admitted to the department of neurology, rheumatology, endocrinology, and nephrology were 492 (50.2%), 354 (36.1%), 113 (11.5%), and 21 (2.1%) respectively.

### 3.2 Characteristics of prophylactic use of PPIs

Of the 980 patients, 271 (27.7%) were treated with PPIs (either alone or in combination), 670 (68.4%) were treated with histamine 2 receptor antagonists (H_2_RAs) (either alone or in combination), 11 (1.1%) were treated with GMPs alone, and 28 (2.9%) were treated with no acid suppressants or GMPs, as shown in Table 1. Omeprazole was prescribed in 63.5% of the patients who were prescribed PPIs, followed by pantoprazole (22.1%), esomeprazole (7.4%), and rabeprazole (7.0%). Among the patients prescribed PPIs, 262 (96.7%) patients received oral PPIs. 181 patients received a standard dose of PPIs once a day, and 90 patients received a standard dose of PPIs twice a day. The average duration of PPIs use was 8.0 (5.0,11.0) days.

**TABLE 1:** The type and proportion of prophylactic use of acid suppressants or GMPs.

Type	N (%)	Type	N (%)
PPIs	225 (23.0)	H_2_RAs	605 (61.7)
PPIs + GMPs	36 (3.7)	H_2_RAs + GMPs	65 (6.6)
PPIs + H_2_RAs	9 (0.9)	GMPs	11 (1.1)
PPIs + H_2_RAs + GMPs	1 (0.1)	No medication	28 (2.9)

*GMPs*, gastric mucosa protectants; *PPIs*, proton pump inhibitors, *H*_*2*_ *RAs*, histamine 2 receptor antagonists.

### 3.3 Influencing factors associated with the prophylactic use of PPIs

Finally, 952 patients who were prescribed acid suppressants or GMPs were divided into PPIs and non-PPIs groups based on whether or not using PPIs. In univariate analysis, 14 factors were significantly associated with prophylactic use of PPIs (*p* < 0.05): age, gender, history of GERD, history of PU, platelet count, number of concomitant diseases, coronary heart disease, cerebral infarction, professional titles of doctors, glucocorticoid dose, antiplatelet agents, immunosuppressants, blood-activating drugs and betahistine (Table 2). The results of multicollinearity analysis showed that VIF values of 14 factors were less than 10. In multiple logistic regression analysis, age ≥80 years, history of GERD, low platelet count, number of concomitant diseases, junior doctors, glucocorticoid dose (higher than 50 mg, measured by methylprednisolone), antiplatelet agents, immunosuppressants, and betahistine were associated with more prophylactic use of PPIs (*p* < 0.05) (Table 3).

**TABLE 2:** Patient demographic and clinical characteristics.

Variable	PPIs group (n = 271)	Non-PPIs group (n = 681)	*P*
Age (years)			<0.001
<65	208 (76.8)	582 (85.5)	
65–79	54 (19.9)	97 (14.2)	
≥80	9 (3.3)	2 (0.3)	
Gender			0.027
Male	140 (51.7)	298 (43.8)	
Female	131 (48.3)	383 (56.2)	
BMI (kg/m^2^)	24.8 (22.9.27.3)	25.4 (23.1.27.4)	0.462
Smoke, currently	58 (21.4)	111 (16.3)	0.063
Alcohol, currently	45 (16.6)	90 (13.2)	0.176
History of GERD	65 (24.0)	88 (12.9)	<0.001
History of PU	7 (2.6)	4 (0.6)	0.024
Serum creatinine (umol/L)	64.0 (52.0.73.3)	62.0 (53.0.73.0)	0.621
INR	0.98 (0.93.1.03)	0.98 (0.93.1.03)	0.972
Platelet count (10^9^/L)	212.0 (179.0.259.0)	225.0 (191.0.270.0)	0.003
Length of hospital stay (days)	11.0 (7.0.15.0)	10.0 (7.0.14.0)	0.191
Number of concomitant diseases	6.0 (3.0.10.0)	4.0 (2.0.7.0)	<0.001
Medical insurance			0.298
Yes	260 (95.9)	642 (94.3)	
No	11 (4.1)	39 (5.7)	
Concomitant diseases			
Diabetes	59 (21.8)	120 (17.6)	0.139
Coronary heart disease	24 (8.9)	29 (4.3)	0.005
Cerebral infarction	26 (9.6)	26 (3.8)	<0.001
Atrial fibrillation	5 (1.8)	4 (0.6)	0.150
Hypertension	97 (35.8)	214 (31.4)	0.195
Hyperuricemia/gout	19 (7.0)	47 (6.9)	0.952
Liver dysfunction	26 (9.6)	69 (10.1)	0.803
Professional titles of doctors			0.001
Junior	105 (38.7)	189 (27.8)	
Middle/Senior	166 (61.3)	492 (72.2)	
Glucocorticoid dose^a^			0.003
≤20	52 (19.2)	167 (24.5)	
≤50, >20	47 (17.3)	148 (21.7)	
<500, >50	39 (14.4)	122 (17.9)	
≥500	133 (49.1)	244 (35.8)	
Duration of glucocorticoid therapy	8.0 (4.0.12.0)	8.0 (6.0.10.0)	0.192
Concomitant medications			
Antiplatelet agents	62 (22.9)	67 (9.8)	<0.001
Anticoagulants	5 (1.8)	5 (0.7)	0.244
NSAIDs	5 (1.8)	15 (2.2)	0.728
Immunosuppressants	94 (34.7)	171 (25.1)	0.003
Anti-infective drugs	31 (11.4)	75 (11.0)	0.850
Selective serotonin reuptake inhibitors (SSRIs)	4 (1.5)	11 (1.6)	1.000
Blood-activating drugs	53 (19.6)	95 (14.0)	0.031
Bisphosphonates	35 (12.9)	66 (9.7)	0.145
Antiepileptic drugs	4 (1.5)	9 (1.3)	1.000
Lumbrokinase	3 (1.1)	11 (1.6)	0.772
Betahistine	7 (2.6)	3 (0.4)	0.01

*PPIs*, proton pump inhibitors; *BMI*, body mass index; *GERD*, gastroesophageal reflux disease; *PU*, peptic ulcer; *INR*, international normalized ratio; *NSAIDs*, nonsteroidal anti-inflammatory drugs; *SSRIs*, selective serotonin reuptake inhibitors.

^a^ Glucocorticoid dose measured by methylprednisolone.

**TABLE 3:** Multiple logistic regression analysis of factors associated with the prophylactic use of PPIs.

Variable	Adjusted OR (95% CI)	*P*
Age (years)		
<65		
65–79	1.160 (0.739–1.821)	0.518
≥80	7.009 (1.424–34.495)	0.017
Gender	1.047 (0.735–1.493)	0.798
Smoke, currently	1.071 (0.685–1.674)	0.764
History of GERD	2.047 (1.338–3.133)	0.001
History of PU	2.982 (0.734–12.112)	0.127
platelet count	0.997 (0.994–0.999)	0.004
Number of concomitant diseases	1.104 (1.056–1.153)	<0.001
Coronary heart disease	0.703 (0.334–1.484)	0.356
Cerebral infarction	1.318 (0.671–2.589)	0.423
Professional titles of doctors	1.755 (1.248–2.468)	0.001
Glucocorticoid dose^a^		
≤20		
≤50, >20	1.764 (1.000–3.114)	0.050
<500, >50	2.455 (1.371–4.395)	0.003
≥500	4.070 (2.474–6.695)	<0.001
Antiplatelet agents	2.567 (1.456–4.524)	0.001
Immunosuppressants	1.477 (1.014–2.153)	0.042
Blood-activating drugs	1.194 (0.735–1.941)	0.473
Betahistine	5.503 (1.124–26.950)	0.035
NSAIDs	1.341 (0.432–4.160)	0.611

*PPIs*, proton pump inhibitors; *GERD*, gastroesophageal reflux disease; *PU*, peptic ulcer; *NSAIDs*, nonsteroidal anti-inflammatory drugs.

^a^ Glucocorticoid dose measured by methylprednisolone.

## 4 Discussion

To the best of our knowledge, this is the first study to explore the current status and influencing factors of prophylactic use of PPIs in internal medicine inpatients receiving glucocorticoid therapy in China. In our study, the rate of prophylactic use of PPIs in internal medicine inpatients receiving glucocorticoid therapy was 27.7%, which was higher than in a previous study (Munson et al., 2012). The previous study used the HealthCore Integrated Research Database, including outpatient patients, whereas our study population was single-hospital internal medicine inpatients. We did not evaluate the rationality of prophylactic use of PPIs, taking into account the lack of precise and objective evaluation criteria and the differences in relevant guidance or consensus statements (Writing Group of Expert Consensus on the Preventive Application of Proton Pump Inhibitors, 2018; Liu et al., 2013; Caplan et al., 2017; Lanza et al., 2009; Collet et al., 2021).

Among the patients prescribed PPIs, 90 (33.2%) patients received a standard dose of PPIs twice a day. Relying on the unique pharmacological effects of PPIs, its acid suppression effect can be maintained for 16–18 h, allowing for prophylactic use once a day (Savarino et al., 2018; Clarke et al., 2022; Liu et al., 2024). In addition, overuse of PPIs is associated with a variety of adverse events in patients, such as pneumonia and Clostridioides difficile infection (Savarino et al., 2018). Therefore, prophylactic use of PPIs twice a day is not appropriate.

In our study, we found 111 patients receiving a combination of acid suppressants and GMPs, including 36 patients receiving a combination of PPIs and GMPs, 9 patients receiving a combination of PPIs and H_2_RAs, and 1 patient receiving a combination of PPIs, H_2_RAs and GMPs. Currently, there is no evidence to support the combination of acid suppressants and GMPs to prevent glucocorticoid-related gastrointestinal adverse reactions. In general, PPIs should not be combined with other acid suppressants (National Health Commission of the People’s Republic of China, 2020). For GERD patients with persistent nocturnal symptoms, H_2_RAs can be added before bedtime on the basis of PPIs treatment (Katz et al., 2022; Yadlapati et al., 2022). Therefore, there is no need to combine PPIs and H2RAs to prevent glucocorticoid-related gastrointestinal adverse reactions.

The risk of PU and gastrointestinal bleeding caused by glucocorticoids alone is very low, so routine use of PPIs to prevent glucocorticoid-related gastrointestinal adverse reactions is not recommended (Caplan et al., 2017; Dorlo et al., 2013). Prophylactic use of PPIs should only be considered if there are high-risk factors for gastrointestinal bleeding or PU in patients taking glucocorticoids (Writing Group of Expert Consensus on the Preventive Application of Proton Pump Inhibitors, 2018; Liu et al., 2013; Caplan et al., 2017; Lanza et al., 2009; Collet et al., 2021). However, there are no guidelines for direct reference at present, mainly referring to the guidelines related to NSAIDs and antithrombotic drugs (Lanza et al., 2009; Collet et al., 2021).

Our study showed that age ≥80 years, history of GERD, and antiplatelet agents were associated with more prophylactic use of PPIs in internal medicine inpatients receiving glucocorticoid therapy. These three factors are known risk factors for gastrointestinal bleeding, which are mentioned in many studies or guidelines (Lanza et al., 2009; Collet et al., 2021; Herzig et al., 2013). Slightly different is that previous studies or guidelines have shown that age older than 65 years is a risk factor for gastrointestinal bleeding, while our study found that 65–79 years of age was not associated with more prophylactic use of PPIs. Of course, glucocorticoid users who only have advanced age as a risk factor do not need to use PPIs. Compared with patients between 65 and 79 years old, patients 80 years and older have more other risk factors for gastrointestinal bleeding, and are more likely to use PPIs.

It is worth mentioning that a history of PU and combined with NSAIDs were not associated with more prophylactic use of PPIs. Experts from Canada suggested that glucocorticoid users with high-risk factors for gastrointestinal bleeding or PU, such as those using NSAIDs or patients with a history of ulcers, should consider prophylactic use of PPIs (Liu et al., 2013). A nested case-control study demonstrated a nearly fourfold increased risk of PU among glucocorticoid users who concurrently received NSAIDs compared with those not using NSAIDs (Piper et al., 1991). The consensus from China also recommended the prophylactic use of PPIs for patients with systemic corticosteroids combined with NSAIDs (Writing Group of Expert Consensus on the Preventive Application of Proton Pump Inhibitors, 2018). On the one hand, our results may be attributed to the small sample size of patients with a history of PU or those using NSAIDs. On the other hand, it also suggests that physicians are not aware of the need for prophylactic use of PPIs in this particular population of glucocorticoid users.

Our study showed that number of concomitant diseases and glucocorticoid dose (higher than 50 mg, measured by methylprednisolone) were associated with more prophylactic use of PPIs, which was consistent with a previous study (Munson et al., 2012). For the former, the authors consider that it may be because the more concomitant diseases the patient has, the greater the risk of gastrointestinal bleeding. We will continue to seek the reasons behind the association between number of concomitant diseases and prophylactic use of PPIs. For the latter, our results also showed that the probability of prophylactic use of PPIs increased with increasing glucocorticoid dose. Tseng et al. discovered that moderate to high dose glucocorticoids were associated with an increased risk of peptic ulcer bleeding and dose-dependent (Tseng et al., 2015). This can also explain our results to some extent.

In our study, immunosuppressants and betahistine were identified as influencing factors for increased prophylactic use of PPIs in glucocorticoid users. The top three immunosuppressants were mycophenolate (49.4%), methotrexate (12.1%), and hydroxychloroquine (10.9%). One of the common adverse effects of immunosuppressants is gastrointestinal effects, especially mycophenolate (Nunes FA and Lucey MR, 1999). However, gastrointestinal ulcers or bleeding induced by immunosuppressants are rare, and there are no reports that the use of immunosuppressants is a risk factor for gastrointestinal ulcers or bleeding. This also applies to betahistine, although this drug has a histamine-like effect and may cause gastric acid secretion by affecting the H receptor. The risk of gastrointestinal ulcers or bleeding caused by glucocorticoids combined with immunosuppressants or betahistine, and whether it is necessary to prophylactic use of PPIs remains to be explored.

To our surprise, junior doctors was associated with more prophylactic use of PPIs. It must be mentioned that a similar result was also found in our previous study (Liu et al., 2024). It is not clear whether this is due to the lack of knowledge among junior doctors. Next, we will conduct a questionnaire survey on the prophylactic use of acid suppressants in patients with glucocorticoids among physicians in our hospital. In addition, low platelet count was a predictor of more prophylactic use of PPIs in our study. The lower the platelet count in patients, the higher the risk of gastrointestinal bleeding. A retrospective cohort study based on Taiwan’s National Health Insurance Research Database also showed that patients with thrombocytopenia had an increased risk of gastrointestinal bleeding compared with those without thrombocytopenia (HR = 2.61; 95% CI, 2.05–3.32) (Lo et al., 2016).

In response to this study’s findings, clinical pharmacists will carry out special training for physicians, especially junior doctors. For patients taking glucocorticoids combined with high-risk factors such as a history of PU, combined with NSAIDs, PPIs should be actively administered. For patients with other factors such as combined immunosuppressants, betahistine, *etc.*, the risk of gastrointestinal bleeding should be comprehensively evaluated, and PPIs should be used individually to ensure rational drug use.

Our study has the following limitations. First, this was a retrospective, single-center study. However, we believe our result is representative based on our hospital scale. Second, we did not evaluate the appropriateness of prophylactic use of PPIs, mainly considering that there are no clear evaluation criteria at present.

## 5 Conclusion

This study confirms that the prophylactic use of PPIs in internal medicine inpatients receiving glucocorticoid therapy is common in China. There are some inappropriate situations, such as the prophylactic use of PPIs twice a day. Additionally, age ≥80 years, history of GERD, low platelet count, number of concomitant diseases, junior doctors, glucocorticoid dose, antiplatelet agents, immunosuppressants, and betahistine are associated with more prophylactic use of PPIs. Clinical pharmacists should take effective measures to promote the rational prophylactic use of PPIs, such as providing specialized training to doctors and conducting multidisciplinary discussions.

## Data Availability

The original contributions presented in the study are included in the article/supplementary material, further inquiries can be directed to the corresponding author.
